# Immunogenicity and Tolerability of a SARS-CoV-2 TNX-1800, a Live Recombinant Poxvirus Vaccine Candidate, in Syrian Hamsters and New Zealand White Rabbits

**DOI:** 10.3390/v15102131

**Published:** 2023-10-21

**Authors:** Mayanka Awasthi, Anthony Macaluso, Scott J. Goebel, Erin Luea, Ryan S. Noyce, Farooq Nasar, Bruce Daugherty, Sina Bavari, Seth Lederman

**Affiliations:** 1 Tonix Pharmaceutical, Frederick, MD 21701, USA; mayanka.awasthi@tonixpharma.com (M.A.); scott.goebel@tonixpharma.com (S.J.G.); farooq.nasar@tonixpharma.com (F.N.); sina.bavari@tonixpharma.com (S.B.); 2 Southern Research, Birmingham, AL 35205, USA; eluea@southernresearch.org; 3 Department of Medical Microbiology & Immunology, Li Ka Shing Institute of Virology, University of Alberta, Edmonton, AB T6G 2R3, Canada; noyce@ualberta.ca; 4 Tonix Pharmaceuticals, Chatham, NJ 07928, USA; bruce.daugherty@tonixpharma.com; 5 Tonix Pharmaceuticals, Dartmouth, MA 02745, USA

**Keywords:** SARS-CoV-2, TNX-1800, vaccine, vaccine platform, horsepox, spike protein, antibody titer, immunogenicity, IgG

## Abstract

TNX-1800 is a preclinical stage synthetic-derived live attenuated chimeric horsepox virus vaccine engineered to express the SARS-CoV-2 spike (S) gene. The objectives of this study were to assess the safety, tolerability, and immunogenicity of TNX-1800 administration in Syrian golden hamsters and New Zealand white rabbits. Animals were vaccinated at three doses via percutaneous inoculation. The data showed that the single percutaneous administration of three TNX-1800 vaccine dose levels was well tolerated in both hamsters and rabbits. At all dose levels, rabbits were more decerning regarding vaccine site reaction than hamsters. Lastly, no TNX-1800 genomes could be detected at the site of vaccination. Post-vaccination, all animals had anti-SARS-CoV-2 spike protein IgG specific antibody responses. These data demonstrate that TNX-1800 infection was limited, asymptomatic, and cleared by the end of this study, and a single dose was able to generate immune responses.

## 1. Introduction

The COVID-19 pandemic, caused by the novel coronavirus SARS-CoV-2, has had a profound impact on global public health. The virus first emerged in Wuhan, China, in late 2019 to cause a global pandemic, leading to more than ~71 million deaths worldwide [1,2,3]. Enormous effort has been dedicated to rapidly generating effective vaccines. Multiple vaccines based on various platforms have been developed, including mRNA [4], live attenuated adenovirus [5], and protein subunit [6]. These vaccines have been rapidly assessed in human clinical trials to ensure their safety and efficacy and have received Food and Drug Administration (FDA) approval. Despite the remarkable success of COVID-19 vaccine development, the global rollout of vaccines has faced significant challenges such as a two-dose regimen, lack of durable immunity against multiple variants, and reliance on the cold chain [7]. As the SARS-CoV-2 infection continues to evolve and with the emergence of new variants, new vaccine platforms are needed to address these outstanding issues. One potential platform that can be utilized to address these issues is poxvirus. Poxvirus such as vaccinia has been shown to be immunogenic by a single dose, induce durable immunity lasting decades, and are not reliant on the cold chain. These characteristics enabled the utilization of Vaccinia virus vaccines to eradicate smallpox [8]. We developed a recombinant chimeric horsepox virus platform, a close relative of the Vaccinia virus, and engineered it to express the SARS-CoV-2 spike gene. One critical step in live attenuated vaccine development is the evaluation of safety and tolerability in small animal models. In this report, we have assessed TNX-1800 safety, tolerability, and immunogenicity, in Syrian golden hamsters and New Zealand white rabbits at three different doses following percutaneous administration. The clinical observations, local Draize scoring, body weights, and viral load in skin samples collected from the injection sites at necropsy showed that there was no disseminated clinical disease, the virus did not persist at the site of infection and the vaccine was well tolerated. Further, a single dose was able to generate an antibody response in hamsters and rabbits.

## 2. Materials and Methods

### 2.1. Vaccine Information

TNX-1800 is a recombinant HPXV [9], expressing the codon-optimized, SARS-CoV-2 Spike gene (Wuhan strain, NC_045512) from a non-essential insertion locus. Expression of the SARS-CoV-2 Spike gene is driven by a synthetic early/late poxvirus promoter. The methods for the generation of the recombinant VACV control vaccine, TNX-1200, have been previously described [10]. Both virus preparations were resuspended in Tris-HCl (10 mM, pH 8.0).

### 2.2. Ethics Statement

This work was supported by an approved Institute Animal Care and Use Committee (IACUC) animal research protocol in compliance with the Animal Welfare Act, PHS policy, and other federal statutes and regulations relating to animals and experiments involving animals. The facility where this research was conducted is accredited by the Association for Assessment and Accreditation of Laboratory Animal Care (AAALAC International) and adheres to principles stated in the Guide for the Care and Use of Laboratory Animals, National Research Council, 2011 [11].

### 2.3. Study Design and Immunization Procedure

Syrian golden hamsters and New Zealand white rabbits aged at least 13 weeks old and weighing ~80–150 g and weighing 2.0 to 3.5 kg, respectively, were obtained. All animals were immunized with three doses (3 × 10^6^, 1 × 10^6^, 3 × 10^5^ PFU) on day 1 via percutaneous inoculation between the shoulder blades. Prior to each dose administration, the dosing site was shaved and marked. The site was sanitized, and 10 μL of either TNX-1200 or TNX-1800 was placed on the skin using a sterile pipette tip. A bifurcated needle was used to push each virus vaccine under the skin by penetrating the skin vertically approximately 15 times. The residual vaccine was removed using sterile gauze.

### 2.4. Body Weight, Lesion Counts, and Draize Scoring

Body weight and detailed clinical observations specifically including evaluations of erythema, edema, and percutaneous (eschar/scab/pox formation) were determined, starting prior to dosing on day 1, and twice weekly. Photos were taken of all lesions and any abnormal skin observations.

### 2.5. Enzyme-Linked Immunosorbent Assay (ELISA)

Serum samples were collected on days 1, 14, 21, and 28. To measure SARS-CoV-2 spike protein antibody responses during the vaccination phase. Briefly, 96-well ELISA plates were coated with 100 μL/well of 0.5 ug/mL SARS-CoV-2 (2019-nCoV) spike S1 + S2 ECD-His Recombinant Protein (Sino Biological, Chesterbrook, PA, USA, Cat no# 40589-V08B1) antigen in phosphate-buffered saline (PBS) and incubated overnight at 4 °C. Serum samples were serially diluted in 5% non-fat milk/0.05% PBST and added at 100 μL/well. Specific antibodies were detected using a goat anti-Hamster IgG (H + L) cross-adsorbed secondary antibody, HRP, Invitrogen (Thermo Fisher Scientific, Waltham, MA, USA, Cat no# PA5-33286), and Goat anti-Rabbit IgG (H + L) cross-adsorbed secondary antibody, HRP, Invitrogen (Thermo Fisher Scientific, Waltham, MA, USA, Cat# 31462). Plates were developed with 100 μL/well of ABTS ((2,2’-Azinobis [3-ethylbenzothiazoline-6-sulfonic acid]-diammonium salt) substrate for 15 to 20 min, stopped with 100 μL of 1% sodium dodecyl sulfate (SDS) in distilled water and read at 450 nm using a SpectraMax plate reader (Molecular Devices, Sunnyvale, CA, USA).

### 2.6. Viral Load by Quantitative PCR (qPCR) Analysis

Viral loads were measured in tissue homogenates of skin punch biopsy samples. Total DNA was extracted from each sample using a QIAcube robot and viral genome copies were quantified via qPCR. Briefly, samples were extracted using TRI Reagent (Sigma Aldrich, St. Louis, MO, USA, Cat no# T9424) following the manufacturer’s recommendations. Viral DNA isolated from samples was eluted with nuclease-free H_2_O and stored at −70 °C or below. The quantitative PCR (qPCR) reaction used to assess vaccine viral load was targeted to the Orthopox E9L DNA Polymerase gene using commercially available Pan-orthopox Virus E9L Gene-specific Quantitative PCR Assay Detection Kit (BEI Resources, Manassas, VA, USA, Cat no# NR-9350).

### 2.7. Statistical Analysis

Comparative statistical analysis of the body weight data and other parameters as deemed appropriate were performed using Provantis^®^ (v10.1). Statistical analysis of other data (e.g., immunology) consisted of descriptive statistics such as mean, standard deviation, and coefficient of variation.

## 3. Results

### 3.1. Post-Dosing Clinical Observations and Vaccine Persistence

**Figure 1:**
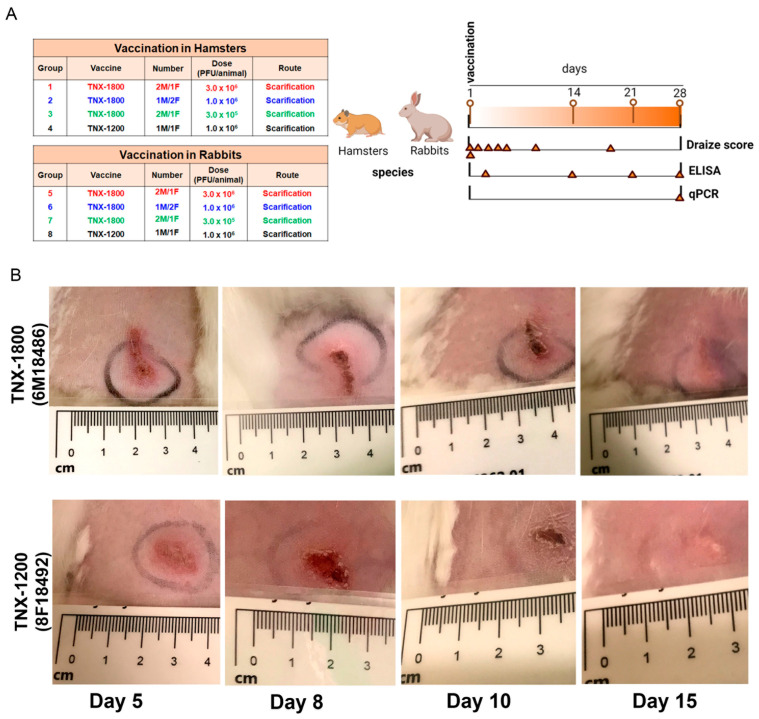
(**A**) Schematic of the hamsters and rabbits study design. Males (M) and females (F) hamsters and rabbits were vaccinated with TNX-1800 (three different dose levels) and TNX-1200. Group 4 and group 8 were used as positive controls for erythema and edema scoring. Following vaccination, Draize score, immunogenicity, and qPCR were performed at intervals indicated by triangles. (**B**) Photographs of the lesions at the injection site of Group 6 (TNX-1800) and group 8 of rabbits (TNX-1200).

To evaluate the safety, tolerability, and immunogenicity of TNX-1800 in hamsters and rabbits, four groups of each animal were studied. The hamsters’ groups 1–3, (*n* = 3 animals in each group) and the rabbits’ groups 5–7, (*n* = 3 animals in each group) were vaccinated on day 1 via percutaneous administration using bifurcated needles with different doses of TNX-1800. Similarly, group 4 of hamsters (*n* = 2) and group 8 of rabbits (*n* = 2) were vaccinated with a single dose of 1.0 × 10^6^ PFU of TNX-1200 (Figure 1A). Following percutaneous dose administration, animals in groups 1–8 were observed for local dermal responses following vaccination. The representative photograph of days 5, 8, 10, and 15 are shown (Figure 1B). Group 4 of hamsters and group 8 of rabbits, vaccinated via percutaneous inoculation with a single dose of 1.0 × 10^6^ PFU of TNX-1200, were used as a comparative control for erythema and edema scoring. All animals in this study consistently maintained or gained body weights and any fluctuations were considered normal for all the animals (Table S1). There were no abnormal clinical observations documented at any dose level for rabbits or hamsters. All hamsters and rabbits exhibited minimal to moderate erythema within 2 h of dosing. Minimal erythema was resolved in five hamsters and one rabbit 5 h post-vaccination. Observations of minimal edema increased in frequency in both species beginning on day 2 and continuing through days 5 and 10 for hamsters and rabbits, respectively. The rabbits at all dose levels consistently had higher grades of erythema and edema than hamsters at the same dose levels (Table S2).

To investigate the potential persistence of TNX-1800 at the vaccination site, we tested the site of the vaccine 4 weeks after TNX-1800 administration for the presence of pox-related genes by qPCR. The assessment of the vaccine-induced viral load by qPCR assay in skin samples collected from the injection sites four weeks post-dosing indicated no detectable levels of viral genomes present in the tissues (Table S2).

### 3.2. TNX-1800 Immunogenicity

**Figure 2:**
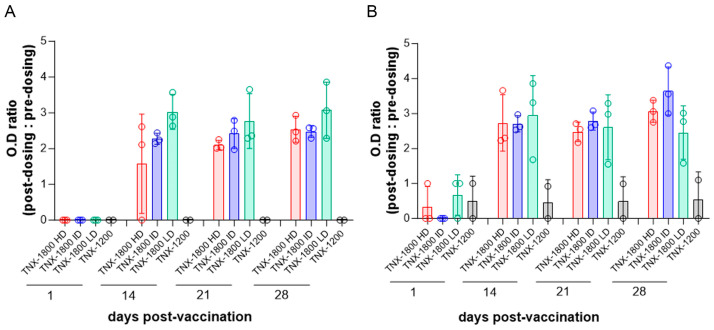
Immunogenicity following vaccination in (**A**) hamsters and (**B**) rabbits as determined by ELISA. The graph depicts the post-dosing to pre-dosing OD ratio corresponding to the total spike-specific IgG. Animals vaccinated with TNX-1800 HD: high dose (3 × 10^6^ PFU/animal), ID: intermediate dose (1 × 10^6^ PFU/animal), LD: low dose (3 × 10^5^ PFU/animal) are represented in red, blue, and green, respectively. The control group vaccinated with TNX-1200 is represented in grey.

Humoral responses in hamster (groups 1–4) and rabbit (groups 5–8) serums were measured by detecting the binding of SARS-CoV-2 spike protein-specific immunoglobulins G (IgG) in an ELISA format. Antibody titers were observed in almost all animals at 14 days post vaccination. In both animal models, all dose groups yielded similar ELISA titers regardless of the dose group. Peak titers were achieved in both species by day 28 post vaccination in most animals (Figure 2A,B).

Hamsters in group 3 (TNX-1800 dose of 3.0 × 10^5^) consistently had higher average OD titer ratios throughout this study (Figure 2A). TNX-1800 vaccinated rabbits consistently had a higher IgG titer value beginning on day 14 when compared to hamsters at the same dose level. Groups 5 and 6 showed the highest average OD titer ratio on day 28: 3.06 and 3.64, respectively. Rabbits in group 7 (TNX-1800 3.0 × 10^5^) had the highest IgG titer ratio on day 14 (2.95) followed by a slight decrease to 2.61 and 2.12 in the following two weeks (Figure 2B). However, these levels of anti-SARS-CoV-2 spike IgG were not statistically different regardless of the dose. As expected, control groups vaccinated with TNX-1200 lacking the SARS-CoV-2 spike gene cassette (groups 4 and 8) had low background levels of anti-SARS-CoV-2 spike-specific IgGs throughout this study.

## 4. Discussion

Multiple vaccine platform approaches have been used to deliver SARS-CoV-2 spike as a vaccine and elicit neutralizing immune responses, including live attenuated viral vectors (e.g., adenovirus), nucleic acids (primarily mRNA), and protein subunits [4,5,6,12,13]. The combination of quickly waning immunity, especially in the upper respiratory system, and the introduction of new SARS-CoV-2 variants with modified spikes has raised concern about the current vaccines [14,15]. Also, accumulating data suggest the currently approved vaccines are not capable of fully protecting against infection/reinfection due to suboptimal mucosal immunity in the upper respiratory tract [16]. Thus, there is a gap in defense against evolving SARS-CoV-2 infection which may be filled by the ongoing need to optimize SARS-CoV-2 vaccines, either by adapting current vaccine strategies or by searching for alternative technologies to overcome hurdlers. Exploring alternative delivery of SARS-CoV-2 spike as a vaccine such as using novel live attenuated poxvirus vectors may provide an additional vector platform to circumvent issues with current approved vaccines.

Here, we tested the tolerability of a single percutaneous administration of a live horsepox vector expressing the Wuhan spike gene and its ability to elicit binding antibodies. TNX-1800 vaccine at all dose levels did not cause disseminated clinical disease throughout the study period. Observations of erythema were recorded beginning at 2 h after administration and edema increased in frequency beginning on day 2. Most local Draize observations were noted through day 10. Rabbits at all dose levels consistently had more dermal observations related to erythema and edema than hamsters at the same dose levels. Post-vaccination viral persistence was assessed by qPCR in skin samples collected at the vaccination site at four weeks and was found to be below the limit of detection for both species in all groups (Table S2). Lastly, all animals generated anti-SARS-CoV-2 spike protein-specific antibody response. These data demonstrate that TNX-1800 infection was limited, asymptomatic, cleared by the end of the study, and was immunogenic following a single percutaneous vaccination.

These encouraging preliminary tolerability and immunogenicity data suggest the potential of this platform for further development of future vaccines against SARS-CoV-2 and other pandemic-causing viruses. Future efforts will include testing immunogenicity and efficacy in non-human primates. Additional experiments will allow a better understanding of neutralizing antibody titers and test the ability of the vector to carry multiple protective antigens inserts against various infectious diseases.

## Data Availability

Not applicable.

## Supplementary Materials

The following supporting information can be downloaded at: https://www.mdpi.com/article/10.3390/v15102131/s1, Table S1: The body weight of rabbits and hamsters monitored throughout the study. Table S2: Draize score and poxvirus gene copy number (*) in rabbits and hamsters monitored during different time points.
