# Supplementary material for: Immunogenicity and Tolerability of a SARS-CoV-2 TNX-1800, a Live Recombinant Poxvirus Vaccine Candidate, in Syrian Hamsters and New Zealand White Rabbits
Source: Viruses. 2023 Oct 21;15(10):2131. doi: 10.3390/v15102131 (PMC10612059; doi:10.3390/v15102131)
Supplement: Supplementary file 1 [file viruses-15-02131-s001.zip › viruses-2633145-supplementary.pdf]

Supplementary Information:

Table S1. The body weight of rabbits and hamsters monitored throughout the study.

Individual Bodyweights - Hamsters

Sex: Both Bodyweight (grams)

Group 1	Day(s) Relative to Start Date								
	1	5	9	12	15	19	22	26	28
18494	116.3	118.0	119.6	119.4	121.0	123.7	125.0	127.0	127.8
18495	128.2	128.0	127.7	126.8	127.0	128.5	128.4	131.0	128.0
18502	118.6	120.8	126.8	130.1	129.6	132.2	132.9	135.0	137.4
Mean	121.03	122.27	124.70	125.43	125.87	128.13	128.77	131.00	131.07
SD	6.31	5.16	4.44	5.48	4.41	4.26	3.96	4.00	5.49
N	3	3	3	3	3	3	3	3	3

Individual Bodyweights - Hamsters

Sex: Both Bodyweight (grams)

Group 2	Day(s) Relative to Start Date								
	1	5	9	12	15	19	22	26	28
18496	125.0	119.6	119.0	119.3	120.0	122.5	124.7	126.0	129.3
18503	131.0	134.2	135.0	135.8	135.5	137.2	137.2	139.0	140.2
18504	135.7	136.5	135.0	133.4	131.0	134.7	138.0	139.0	140.0
Mean	130.57	130.10	129.67	129.50	128.83	131.47	133.30	134.67	136.50
SD	5.36	9.17	9.24	8.91	7.97	7.87	7.46	7.51	6.24
N	3	3	3	3	3	3	3	3	3

Individual Bodyweights - Hamsters

Sex: Both Bodyweight (grams)

Group 3	Day(s) Relative to Start Date								
	1	5	9	12	15	19	22	26	28
18497	128.0	127.5	125.0	126.9	128.0	128.4	128.3	127.0	127.3
18498	111.5	110.1	109.5	110.3	107.0	110.2	109.0	112.0	113.0
18505	128.5	126.1	126.8	126.4	127.4	128.0	128.5	129.0	131.4
Mean	122.67	121.23	120.43	121.20	120.80	122.20	121.93	122.67	123.90
SD	9.67	9.67	9.51	9.44	11.95	10.39	11.20	9.29	9.66
N	3	3	3	3	3	3	3	3	3

Individual Bodyweights - Hamsters

Sex: Both Bodyweight (grams)

Group 4	Day(s) Relative to Start Date								
	1	5	9	12	15	19	22	26	28
18499	137.0	136.3	139.2	138.8	140.6	139.7	140.0	142.0	142.0
18506	122.7	123.6	120.6	120.2	-	117.1	117.0	120.0	120.0
Mean	129.85	129.95	129.90	129.50	140.60	128.40	128.50	131.00	131.00
SD	10.11	8.98	13.15	13.15	-	15.98	16.26	15.56	15.56
N	2	2	2	2	1	2	2	2	2

Individual Bodyweights - Rabbits

Sex: Both Bodyweight (kg)

Group 5	Day(s) Relative to Start Date								
	1	5	9	12	15	19	22	26	28
18480	3.2	3.2	3.1	3.2	3.2	3.3	3.3	3.4	3.3
18481	2.8	3.0	3.1	3.1	3.1	3.1	3.2	3.1	3.2
18487	2.3	2.4	2.5	2.6	2.6	2.7	2.7	2.7	2.7
Mean	2.77	2.87	2.90	2.97	2.97	3.03	3.07	3.07	3.07
SD	0.45	0.42	0.35	0.32	0.32	0.31	0.32	0.35	0.32
N	3	3	3	3	3	3	3	3	3

Individual Bodyweights - Rabbits

Sex: Both Bodyweight (kg)

Group 6	Day(s) Relative to Start Date								
	1	5	9	12	15	19	22	26	28
18486	-	3.2	3.2	3.3	3.3	3.3	3.3	3.4	3.2
18488	2.5	2.6	2.8	2.8	2.9	3.0	3.1	3.1	2.9
18489	2.5	2.6	2.7	2.8	2.8	2.9	2.9	2.9	2.8
Mean	2.50	2.80	2.90	2.97	3.00	3.07	3.10	3.13	2.97
SD	0.00	0.35	0.26	0.29	0.26	0.21	0.20	0.25	0.21
N	2	3	3	3	3	3	3	3	3

Individual Bodyweights - Rabbits

Sex: Both Bodyweight (kg)

Group 7	Day(s) Relative to Start Date								
	1	5	9	12	15	19	22	26	28
18483	3.1	2.9	3.0	3.1	3.1	3.1	3.2	3.3	3.3
18484	3.0	2.1	3.2	3.2	3.2	3.3	3.3	3.2	3.1
18490	2.5	2.6	2.7	2.7	2.7	2.8	2.8	2.8	2.8
Mean	2.87	2.53	2.97	3.00	3.00	3.07	3.10	3.10	3.07
SD	0.32	0.40	0.25	0.26	0.26	0.25	0.26	0.26	0.25
N	3	3	3	3	3	3	3	3	3

Individual Bodyweights - Rabbits

Sex: Both Bodyweight (kg)

Group 8	Day(s) Relative to Start Date								
	1	5	9	12	15	19	22	26	28
18485	3.0	3.0	3.1	3.2	3.2	3.2	3.2	3.2	3.2
18492	2.4	2.5	2.6	2.7	2.7	2.8	2.8	2.8	2.7
Mean	2.70	2.75	2.85	2.95	2.95	3.00	3.00	3.00	2.95
SD	0.42	0.35	0.35	0.35	0.35	0.28	0.28	0.28	0.35
N	2	2	2	2	2	2	2	2	2

Table S2. Draize score and poxvirus gene copy number (*) in rabbits and hamsters monitored during different time points.

Species			Day 1 (2 hr post)		Day 1 (5 hr post)		Day 2		Day 3		Day 4		Day 5		Day 10		Day 20		Day 28 *
	Group	Animal	Erythema	Edema	Erythema	Edema	Erythema	Edema	Erythema	Edema	Erythema	Edema	Erythema	Edema	Erythema	Edema	Erythema	Edema	Mean Copy number
Hamsters	1	18494	2	0	2	0	2	0	2	0	0	1	1	1	1	0	0	0	0
	1	18495	1	0	1	0	1	0	1	0	1	0	1	0	1	0	0	0	0
	1	18502	1	0	1	0	1	0	1	0	1	0	1	1	1	0	0	0	0
	2	18503	1	0	1	0	1	0	1	0	1	0	1	0	0	0	0	0	0
	2	18504	1	0	0	0	1	0	1	0	0	0	1	0	0	0	0	0	0
	2	18496	2	0	2	0	2	0	1	0	1	0	2	0	1	0	0	0	0
	3	18497	1	0	0	0	0	1	1	0	1	0	1	0	1	0	0	0	0
	3	18498	1	0	0	0	0	0	0	0	1	0	0	1	0	0	0	0	0
	3	18505	1	0	0	0	0	0	0	0	0	1	1	0	0	0	0	0	0
	4	18499	1	0	1	0	1	0	1	0	1	0	2	0	1	0	0	0	0
	4	18506	1	0	0	0	0	1	0	0	0	1	0	0	0	0	0	0	0
Rabbits	5	18480	0	1	0	0	0	1	0	0	0	1	2	1	1	0	1	0	0
	5	18481	2	0	2	0	1	0	1	0	2	1	2	1	2	1	1	0	0
	5	18487	0	0	0	0	0	0	0	1	2	0	2	0	1	1	1	0	0
	6	18488	1	0	0	0	0	1	0	1	0	1	0	1	0	1	0	0	0
	6	18489	1	0	1	0	0	0	0	0	0	1	0	1	1	1	0	0	0
	6	18486	2	0	2	0	1	0	1	0	2	1	2	2	2	0	1	0	0
	7	18483	2	0	2	0	2	0	1	0	1	1	2	0	2	1	1	0	0
	7	18484	1	0	1	1	1	1	0	0	0	1	2	0	2	0	1	0	0
	7	18490	1	1	1	1	1	1	1	1	0	1	1	0	0	1	2	0	0
	8	18485	1	0	1	0	1	1	1	1	2	2	3	1	3	1	1	0	0
	8	18492	1	1	1	1	0	1	0	1	2	0	2	1	3	0	1	0	0
